# Measuring and validating concentrations of steroid hormones in the skin of bottlenose dolphins (*Tursiops truncatus*)

**DOI:** 10.1093/conphys/coaa032

**Published:** 2020-09-08

**Authors:** Thea Bechshoft, Andrew J Wright, Bjarne Styrishave, Dorian Houser

**Affiliations:** 1 Department of Bioscience, Aarhus University, Frederiksborgvej 399, P.O. Box 358, 4000 Roskilde, Denmark; 2 Ocean and Ecosystem Sciences Division, Maritimes Region Fisheries and Oceans Canada, Bedford Institute of Oceanography, 1 Challenger Dr., PO Box 1006, Dartmouth, NS B2Y 4A2, Canada; 3 Department of Environmental Science and Policy, George Mason University, 400 University Drive, Fairfax, VA 22030, USA; 4 Toxicology and Drug Metabolism Group, Department of Pharmacy, Faculty of Health and Medical Sciences, University of Copenhagen, Universitetsparken 2, 2100 Copenhagen OE, Denmark; 5 National Marine Mammal Foundation, 2240 Shelter Island Drive Suite 200, San Diego, CA 92106, USA

**Keywords:** Aldosterone, cetacean, corticosterone, cortisol, DHEA, progesterone, skin, steroid hormones, Stress test, testosterone

## Abstract

A previously published analytical method demonstrated the quantification of the hormone cortisol in cetacean skin. However, little is known about the transfer of hormones between blood and skin. Recognizing that such information is essential to effectively using skin samples within marine mammal stress research, the primary goals of this study were to (i) expand on the number of steroid hormones proved quantifiable in the cetacean skin matrix and (ii) validate the use of cetacean skin as a matrix for measuring stress-related hormones. Five adult bottlenose dolphins were subjected to an out of water stress test. Non-invasive sloughed skin samples were collected from each dolphin: once ~3 and once ~1 week prior to the stress test; at the time of the stress test; and twice weekly for 11 to 17 weeks subsequent to the stress test. LCMS/MS analysis of the samples recovered consistent data on three corticosteroids (cortisol, aldosterone, corticosterone), two androgens (testosterone, DHEA) and one progestagen (progesterone). A range of other hormones were also quantifiable, although not consistently so across samples. Results demonstrated that the hormonal response to an acute stressor could be detected in skin: the time from stress test to skin cortisol peak was an average of 46 days, whereas it was 55 days for corticosterone and 47 days for aldosterone. Results also showed that baseline hormonal concentrations were obtainable from skin samples collected during or immediately after the animals were subjected to the acute stressor. This study further develops and validates a non-invasive method for measuring cortisol and other hormones related to stress, health, and reproduction in the skin of cetaceans, potentially supporting investigations of acute and chronic stress, such as cetacean endocrine responses to distinct (e.g. naval sonar exposure) or prolonged stressors (e.g. shipping noise).

## Introduction

Marine mammals face a wide range of anthropogenic stressors including pollution, underwater noise and climate change (Wright 2009; Maxwell *et al*. 2013; Jenssen *et al*. 2015). When stressors combine to create a chronically stressful situation, they potentially influence the health and reproduction of marine mammals in various ways, including impairing immune function, increasing mortality and reducing reproductive success or effort (Fair and Becker 2000; St. Aubin 2001). While cortisol has long been the preferred biomarker for assessing stress levels in mammals (St. Aubin and Dierauf 2001), it is becoming increasingly clear that other hormones such as aldosterone also play a role in the cetacean stress response (Burgess *et al*. 2017; Champagne *et al*. 2018). Furthermore, other hormones related to life history variables such as age, body condition, and reproductive status may have an effect on the stress response and should be accounted for in studies evaluating markers of stress and their potential physiological impacts (Thomson and Geraci 1986; Mashburn and Atkinson 2004; Schmitt *et al*. 2010; Fair *et al*. 2014; Hart *et al*. 2015).

Blood has long been considered to be the standard source of stress-related hormonal biomarkers e.g. St. Aubin and Dierauf (2001). However, short-term fluctuations arising from natural diel and seasonal cycles (Barrell and Montgomery 1989; Suzuki *et al*. 2003; Oki and Atkinson 2004), as well as capture- and handling-related acute stress (Eskesen *et al*. 2009; Champagne *et al*. 2012; Cook 2012), can potentially overshadow more subtle chronic signals. This fact, together with the difficulties of acquiring blood in free-ranging marine mammals, has led to a series of investigations into other potential matrices for quantifying stress hormones, including vibrissae, baleen, hair, skin, whale blow, saliva, urine and faeces (Constable *et al*. 2006; Pedernera-Romano *et al*. 2006; Hogg *et al*. 2009; Bechshoft *et al*. 2011; Bechshoft *et al*. 2012; Rolland *et al*. 2012; Hunt *et al*. 2013; Bechshoft *et al*. 2015; Hunt *et al*. 2016; Keogh *et al*. 2016; Karpovich *et al*. 2017). While blow, saliva, urine and faeces reflect hormone levels over a time frame of minutes to hours (Cook 2012; Champagne *et al*. 2018), the keratinous (or in case of skin, lipokeratinocytic; Menon *et al*. 1986, Reeb *et al*. 2007) tissues such as vibrissae, baleen, hair and skin reflect the hormone status over a span of multiple weeks to several years, as the various hormones are incorporated into them as they grow (e.g. Noël *et al*. 2014; Hunt *et al*. 2016). Baleen, for example, can reflect up to at least 9 years of fluctuating hormone concentrations, depending on the length of the baleen plates (Hunt *et al*. 2016). Nevertheless, although new studies have made progress in furthering our understanding of the precise time course and extent to which hormones are released into some of these alternative matrices (Hunt *et al*. 2016; Champagne *et al*. 2018), much research is still needed to allow matrices other than blood to be used to their full potential within marine mammal stress research (Atkinson *et al*. 2015).

Cetacean epidermis consists of three histologically distinct layers: *stratum basale* (generating new cells), *s. intermedium* or *spinosum* and *s. externum* or *corneum* (the outermost layer) (Geraci *et al*. 1986). On the underside of the epidermis are rete ridges, interspaced with dermal papillae reaching up as finger-like projections from the underlying dermis (Parry 1949). In bottlenose dolphins, the *S. corneum* is sloughed 12 times per day—however, due to the convoluted structure of the *s. basale*, the transit time for new cells to come to the skin surface is slow (Hicks *et al*. 1985). The deposition rate of serum hormones in cetacean skin has yet to be assessed. Given available assessments of cetacean epidermal turnover rates (70–75 days: Hicks *et al*. 1985, bottlenose dolphin, *Tursiops truncatus*, and St. Aubin *et al*. 1990, beluga, *Delphinapterus leucas*), the acute stress potentially caused by any sampling technique would not be anticipated to enter this matrix for days or weeks. Thus, in a manner that is also seen in other keratinous matrices, a full-depth skin sample would reflect chronic levels, while a thinner section of skin (such as e.g. the sloughed epidermis) taken at the right time relative to the stress exposure would reflect acute levels. However, while the time course and extent of hormonal uptake from the bloodstream into blubber (≤1–2 h) has been examined in cetaceans (Trego *et al*. 2013; Kellar *et al*. 2015; Champagne *et al*. 2017), little is currently known about the transfer of hormones between blood and skin epidermis. Only with such information will skin and other alternative matrices be truly useful in future studies of cetacean stress. Accordingly, our goals in this study were to build on the initial work by Bechshoft *et al*. (2015) by (i) validating the use of cetacean skin as a matrix for measuring cortisol and other stress-related hormones and (ii) expanding the method to encompass a wider range of steroid hormones (stress-related as well as non-stress related). Finally, we aimed to determine baseline levels of the hormones in the skin and to assess their response to a stress test and the following recovery period.

## Methods

### Study animals and experimental design

One female (ID = BLU) and four male (IDs = TRO, NEH, COL, TYH) bottlenose dolphins (aged 13–49 years; mean mass: 202 kg) under the care of the U.S. Navy Marine Mammal Program (MMP) at the Naval Information Warfare Center (NIWC) Pacific in San Diego, CA, USA, participated in this study. Dolphins were housed in open-water netted enclosures within San Diego Bay and experienced normal fluctuations in environmental conditions including day/night cycle, weather and water temperature. Dolphins were fed a fish diet commensurate with their body size as prescribed by each dolphin’s attending veterinarian. The study followed a protocol approved by the Institutional Animal Care and Use Committee of NIWC Pacific, and the Navy Bureau of Medicine and Surgery, and followed all applicable U.S. Department of Defense guidelines for the care of laboratory animals.

The samples for this study were collected in parallel with the performance of a stress test, the design of which is detailed in Champagne *et al*. (2018). Briefly, each dolphin was asked to voluntarily leap out of the water onto a padded tray. Immediately following the voluntary ‘beaching’, a needle was inserted into the peduncle of the dolphin and blood collected every 15 minutes for 2 h while small blubber biopsies were collected hourly (as detailed in Champagne *et al*. 2018). The 2-h stress test significantly raised both cortisol (in serum, blubber, and faeces) and aldosterone (in serum) above baseline concentrations, which indicated an acute stress response (Atkinson *et al*. 2015; Champagne *et al*. 2018). All stress tests and tissue samples related to the stress tests were collected over the course of 2 months (July and August, 2014) to minimize seasonal hormone variability.

Skin was always collected before any other tissue or blood samples, so as to avoid any potential loss of sloughed skin caused by other collection-related manipulations. The collection method was as described in Bechshoft *et al*. (2015), albeit with one minor modification: in short, each sample was collected by moving a rubberized flat edge along the flank of the dolphin twice to collect loose skin that is naturally lost through sloughing (Hicks *et al*. 1985). Skin samples were collected multiple times from each of the five animals, always while they were voluntarily beached: once ~3 weeks (range: 20–23 days) and once ~1 week (range: 6–9 days) prior to the stress test; at the time of the stress test (immediately after the voluntary beaching, but before any other manipulations); and twice weekly for 11 (*n* = 4) to 17 (*n* = 1) weeks subsequent to the stress test. In the post-stress test sampling, the animals were beached only for the purpose of collecting the skin samples. Beaching like this is a daily routine for the animals and they are quite accustomed to it—on its own, the beaching does not cause a stress response (D. Houser, pers. com.), Collectively, it is the blood sampling, blubber sampling, being surrounded by personnel, and the long duration of beaching that combine to make the stress test ‘stressful’. Once collected, samples were frozen (−80°C at the sampling facility; −20°C at the laboratory) until the time of analysis.

The skin sampling regime was established because no literature exists informing the time course over which cortisol is incorporated into dolphin skin. If driven by perfusion, two samples per week would presumably provide the temporal resolution necessary to resolve a more rapid flux of hormone. However, if the process is driven by epidermal turnover (70–75 days: Hicks *et al*. 1985, bottlenose dolphin, and St. Aubin *et al*. 1990, beluga, *Delphinapterus leucas*), samples would need to occur over a longer period of time (at least 11 weeks post-test) to ensure that the peak in skin cortisol concentrations was captured. Hence, sampling twice a week for 11 weeks after the stress test would allow potential validation of the skin cortisol method regardless of whether the measured concentrations were transported to the skin surface as a result of perfusion or skin cell growth. In an attempt to estimate how long it took for the cortisol concentrations to return to baseline, one individual was sampled an additional 6 weeks (i.e. 17 total weeks) after the stress test.

### Steroid hormone analysis

The method implemented in the present study was based on the work by Bechshoft *et al*. (2015), in which cortisol was extracted and analyzed from harbour porpoise skin. We then adapted this method based on Weisser *et al*. (2016) to allow simultaneous analysis of several steroids in the skin using LCMS/MS technology.

### Chemicals

Progesterone (PROG), dehydroepiandrosterone (DHEA), testosterone (TS), aldosterone (ALDO), cortisol (COR) and corticosterone (COS) were all purchased from Sigma-Aldrich, Glostrup, Denmark, with a purity > 96%. Deuterated analogues were applied as internal standards (IS); d8-corticosterone (COSd8) was obtained from CDN isotopes (Pointe-Claire, QC, Canada), while d9-progesterone (PROGd9) d3-testosterone (TSd3), and d4-cortisol (CORd4) were purchased from TRC, all with a deuterated purity above 98%. d6-Dehydroepiandrosterone (DHEAd6) was obtained from Sigma-Aldrich with a deuterated purity > 98%. All utilized solvents were of analytical grade. Methanol was obtained from Fisher Scientific (Leics, UK), acetone and n-heptane were obtained from Fisher Scientific (Slangerup, Denmark). Formic acid 98–100% was purchased from Merck (Merck KGaA, Darmstadt, Germany). All H_2_O used was ultrapure water produced by a Milli-Q system (Millipak 40).

### Standard solutions

For each steroid and deuterated steroid, individual stock solutions of 10.0 μg/mL in methanol were prepared as working solutions. A mixed stock solution was prepared in methanol containing 20 μg/mL of each analyte. A dilution series was then made ranging from 0.0001 to 1 μg/mL in methanol, to be used during validation and application. The internal standard (IS) mixture, containing the five deuterated analogues, was prepared with a concentration of 0.1 μg/mL in methanol.

### PLE procedure

Samples were collected from the scrapers and weighed prior to and after 24 h of lyophilisation using a freeze drier (FD3, Heto Lab Equipment, Allerød, Denmark) operating a −49° and 10^−5^ bar. Steroids were then extracted according to Bechshoft *et al*. (2015) using an ASE 200 pressurized liquid extraction (PLE) system from Dionex (Sunnyvale, California, USA). In short, 22 mL PLE cells were packed with 1 g activated silica gel (particle size 0.063–0.100 mm mesh, Merck, Darmstadt, Germany) and 1 g diatomaceous earth (DE, Dionex, Sunnyvale, CA, USA). The samples were then mixed with 1 g activated DE and ground in a mortar before being added to the cells. Silica gel and DE were activated for 24 h at 105°C before use. For each cell, a single cellulose filter (size 19.8 mm, Dionex, Sunnyvale, CA, USA) was applied at the bottom and at the top of the cell. To each sample, 50 μL IS mixture was applied to the top cellulose filter, corresponding to 5 ng of each IS. Steroids were extracted using a 1:1 (v/v) mixture of methanol and acetone mixture at 1500 psi and 80°C with a 5-min pre-heat followed by two 5-min static extraction cycles with 60% flush volume and 120-sec purge period. PLE extracts were then evaporated close to dryness (~1–2 mL left in the vials) at 60°C under a gentle stream of N_2_ with circular motions and diluted to a total volume of 9 mL using H_2_O.

### Off-line SPE clean-up of extracts

The crude PLE extracts were subjected to further solid-phase extraction (SPE) and clean-up using C18 SPE cartridges. Initially, each SPE column (500 mg Bond elut C18 solid phase extraction cartridges with 10 mL reservoir and 40 µm particle size, Agilent, USA) was placed on a vacuum manifold (IST VacMaster, Biotage, Uppsala, Sweden) and pre-conditioned with 3 mL n-heptane, 3 mL acetone, 3 mL methanol and finally with 5 mL H_2_O with a flow of ~1 mL/min.

Hereafter, the PLE extracts were transferred to the pre-conditioned SPE cartridges. Enrichment was performed at a flow of ~1 mL/min. After enrichment, the SPE cartridge was washed with 9 mL H_2_O at a flow of ~10 mL/min followed by an additional wash under gentle vacuum with 9 mL H_2_O:methanol 75:25 (v/v) solution at a flow of 10 mL/min. Subsequently, steroids were eluted from the SPE cartridges using 5 mL H_2_O:methanol 20:80 (v/v) at a flow of ~1 mL/min. Finally, the collected extract was evaporated to 1 mL under a gentle stream of nitrogen at 60°C and transferred to a 1.5-mL LC-vial.

### Online clean-up in combination with liquid chromatography–tandem mass spectrometry

For online clean-up and chromatographic separation of steroids, a binary 1290 Agilent Infinity Series system and a binary 1100 Agilent HPLC pump were used in combination (Agilent Technologies, Palo Alto, CA, USA). For online clean-up, a C18 enrichment column (μBondapak® C18, 3.9 × 20 mm, 10 μm, Waters) was used. The LC set-up is described in details in Weisser *et al*. (2016). The gradient mobile phase A and B was composed of H_2_O with 0.1% formic acid (v/v) and pure methanol, respectively. The elution gradient was maintained at 10% B for the first 2 min, 10.0–30.0% B from 2.0 to 2.2 min, 30.0–60.0% B from 2.2 to 8.0 min, maintained at 60.0% B from 8.0 to 10.0 min, 60.0–85.0% B from 10.0 to 12.30 min and 85.0–99.5% B from 12.3 to 12.5 min and held at 99.5% B from 12.5 to 14.8 min before re-equilibrating the column. The total run-tine was 16 min. The TTC switching valve directed the flow to waste from 0 to 5.5 min and from 12.5 to 16 min. The temperature of the column oven was set at 40°C, and that of the autosampler was set at 7°C. An injection volume of 100 μL was achieved by installing an 80-μL needle seat and injecting 20 μL into it in four sequential boluses before injecting the entire sample with an injection of the last 20 μL.

### Mass spectrometry

For detection, an AB SCIEX 4500 QTRAP mass spectrometer (Applied Biosystems, Foster City, CA, USA) equipped with electrospray ionization (ESI) was used. Multiple reaction monitoring (MRM) was performed in positive mode during analysis with a target scan time of 0.8 s. The nebulizer current was set at 3 mA with a source temperature of 550°C. Nitrogen was applied as the curtain, collision and ion source gas with settings of 40 psi, high and 40 psi, respectively. LC and MS optimization was conducted using Analyst v. 1.6.2 software package (AB SCIEX) and outputs were processed in MultiQuant v. 3.0 software (AB SCIEX). Calculations and graphics were performed using Microsoft Office Excel 2010 and GraphPad Prism v. 6.03 (GraphPad Software, San Diego, CA, USA).

### Quality criteria

Quality criteria for the method are described detailed in Bechshoft *et al*. (2015). The quality criteria include limit of detection (LOD) and limit of quantification (LOQ), precision, accuracy and relative and absolute recovery for each steroid. Blank and spiked procedural controls followed each sample batch. For each steroid, a nine-point calibration curve with solution concentrations ranging from 0.01–100 ng/mL was used for quantification and to ensure linearity of the method.

### Statistical analyses

Spearman’s correlation tests were used to assess potential correlations between hormone concentrations and sample size or number of samples pooled in one analytical sample, as well as whether sample size was related to Julian day or to the number of days since the previous sample was taken. All statistical analyses were conducted using R (version 3.0.2.39; R Development Core Team 2008) with statistical significance set to *P* ≤ 0.05.

## Results

### Samples

A total of 135 skin samples were collected (BLU = 25, COL = 25, NEH = 25, TRO = 25, TYH = 35), with a mean sample size across all five individuals of 18.2 mg dry weight (dw, S.D. = 8.8; range: 11.0–32.5). Sample mass (in samples weighing ≥50 mg dw, which was the required analytical minimum), was not related to any of the measured hormones (0.12 ≤ *P* ≤ 0.36, −0.30 ≤ *r* ≤ −0.21). Further, in no dolphin was sample size related to Julian day (0.07 ≤ *P* ≤ 1.00, −0.31 ≤ *r* ≤ 0.33) or to the number of days since the previous sample was taken (0.18 ≤ *P* ≤ 0.80, −0.08 ≤ *r* ≤ 0.53). Given the lack of dependence on sample mass, Julian day, or number of days since collection of the previous sample, we pooled a number of the small samples to obtain the sample mass needed to achieve the desired measurement accuracy for this study (given the primary study objective was method validation). Hormone concentrations were not dependent on the number of skin scrape samples pooled into one analytical sample (0.21 ≤ *P* ≤ 0.98, −0.23 ≤ *r* ≤ 0.01). Each pooled sample consisted of individual samples that were collected over the span of 1–20 days (*n* = 41 aggregate samples; mean number of days combined for each sample = 7; S.D. = 5.6; Fig. 1a–f). To undertake statistical analysis, it was necessary to create two subsets of data to account for the different temporal periods represented by the pooled and single samples that had been analyzed: one consisting of only pooled samples that were collected over a maximum of 7 days (*n* = 29 aggregate samples; mean number of days combined for each sample = 4; S.D. = 2.3; Fig. 1g–l) and one consisting of samples from COL, BLU and NEH only, the three individuals whose post-stress test samples represented the finest temporal resolution following sample combination (COL: *n* = 10 aggregate samples, mean number of days combined for each sample = 2.4; BLU: *n* = 6 aggregate samples, mean number of days combined for each sample = 4.8 days; NEH: *n* = 6 aggregate samples, mean number of days combined for each sample = 6.7; Fig. 2).

**Figure 1:**
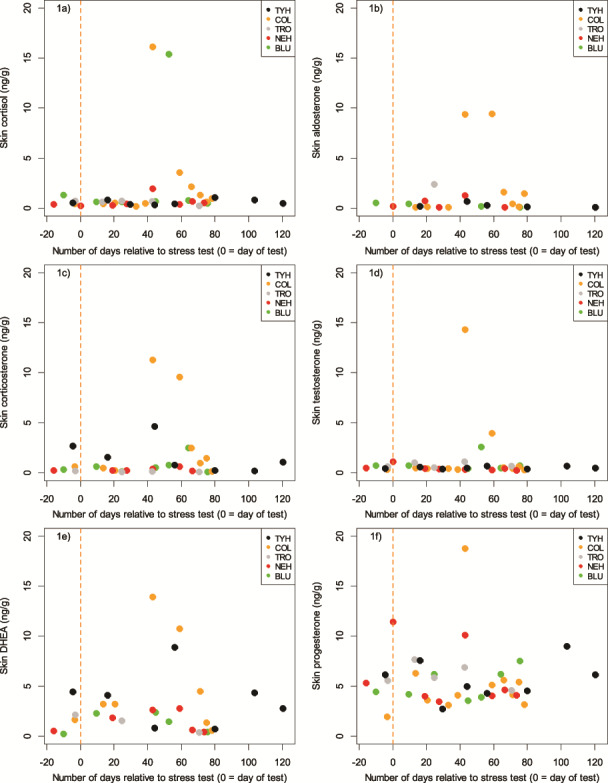
**a–l.** Concentrations of hormones extracted from skin of five bottlenose dolphins, with days relative to the stress test given as the central point in time between the extremes of any pooled samples. a–f show all available data points, each representing skin samples collected over the course of 1–20 days (*n* = 41 aggregate samples; mean number of days combined for each sample = 7; S.D. = 5.6). g–l show the data points which were collected over a maximum of 7 days (*n* = 29 aggregate samples; mean number of days combined for each sample = 4; S.D. = 2.3)

**Figure 1:**
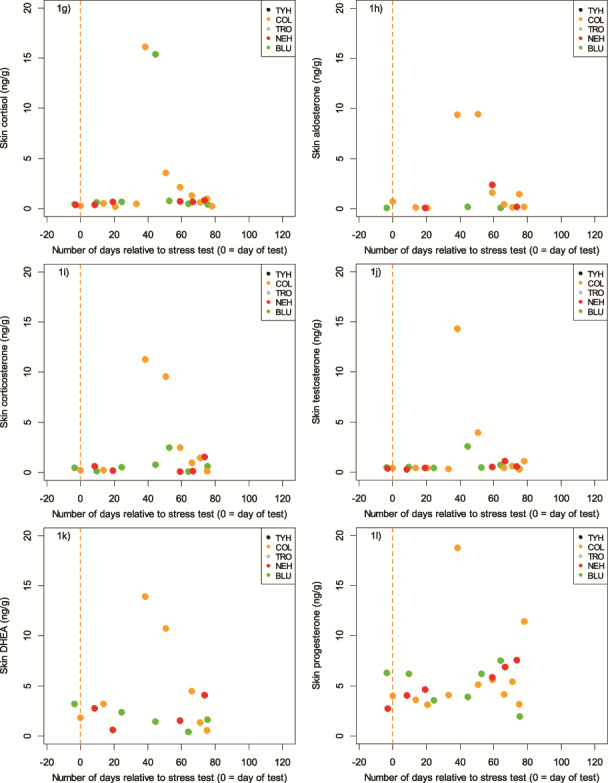
Continued

**Figure 2:**
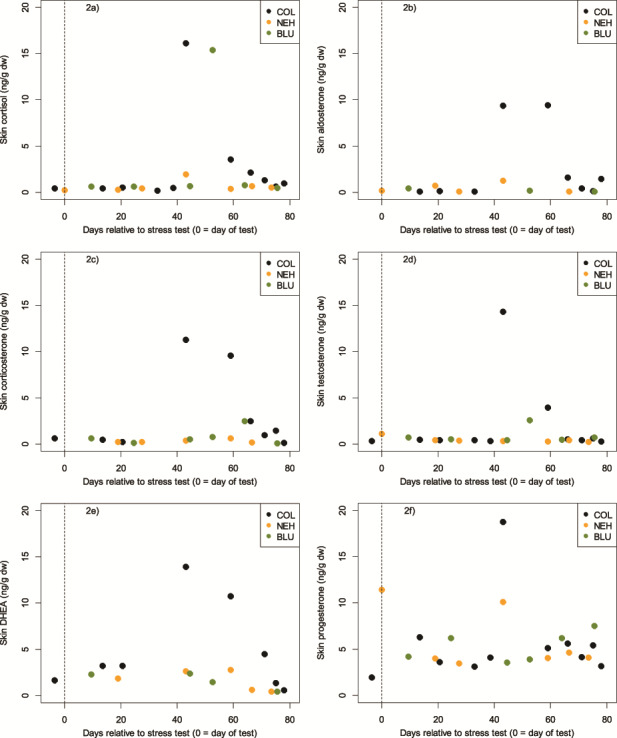
**a–f.** Concentrations of hormones extracted from skin of the three bottlenose dolphins whose samples represented the finest temporal resolution following sample combination (COL: *n* = 10 aggregate samples, mean number of days combined for each sample = 2.4; BLU: *n* = 6 aggregate samples, mean number of days combined for each sample = 4.8 days; NEH: *n* = 6 aggregate samples, mean number of days combined for each sample = 6.67)

### Hormone recovery

A number of hormones were quantifiable in the pooled skin samples (*n* = 41). Consistent data was recovered on three corticosteroids (cortisol, aldosterone and corticosterone), two androgens (testosterone and DHEA) and one progestagen (progesterone; Table 1). Additionally, 11-deoxycortisol, cortisone, estrone and androstenedione (and also trace elements of a fish hormone, 11-ketotestosterone) were quantifiable, although not consistently so across samples.

### Corticosteroids

**Figure 3:**
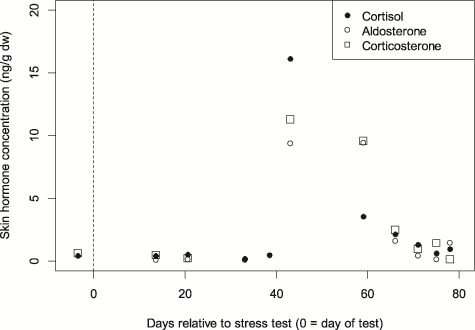
Concentrations of the corticosteroid hormones cortisol, aldosterone, and corticosterone from a single bottlenose dolphin (COL) after being subjected to a stress test

Cortisol was quantifiable in 95.1% of the pooled samples, corticosterone in 80.5% and aldosterone in 58.5%. Hormone concentrations in samples taken prior to as well as immediately following the stress test were comparable, also to the pooled sample most closely following the stress test. We thus considered these samples to represent the baseline concentrations, which across all animals were in the range of, for cortisol, 0.31–0.96 ng/g dw, while peak concentrations (across the remaining aggregated samples) ranged from 1.05–16.17 ng/g dw; for corticosterone, a baseline of 0.16–2.12 ng/g dw, and maximum concentrations in animals with a discernable peak (BLU, COL, NEH, TYH) from 0.61–11.31 ng/g dw; and for aldosterone, 0.09–0.46 ng/g dw, and maximum concentrations in animals with a discernable peak (COL, NEH) from 1.28–9.45 ng/g dw. Minimum skin cortisol concentrations were consistently low and similar across all five animals (Table 1, Fig. 1). However, the magnitude of the response in skin cortisol following the stress test varied significantly between individuals (Table 1 (maximum range), Fig. 1). The results of the three corticosteroids were plotted for the three animals with the highest temporal resolution (COL, NEH and BLU) to assess the lag from the stress test (Fig. 2). Cortisol in these three animals peaked at Days 43, 43 and 53, corticosterone at Days 43, 59 and 64 and aldosterone at Days 43–59 and 43. Based on this, the time from acute stress event to skin cortisol peak in bottlenose dolphins was an average of 46 days or 6.6 weeks, whereas it was 55 days or 7.9 weeks for corticosterone, and 47 days or 6.7 weeks for aldosterone. The ‘peak’ sample for COL consisted of a single sample and shows how the three corticosteroids appear to follow the same pattern relative to the stress test (Fig. 3).

**Table 1:** Hormone concentrations measured in sloughed skin from five bottlenose dolphins (*Tursiops truncatus*)

**Individual**	**Cortisol**		**Aldosterone**		**Corticosterone**		**Testosterone**		**DHEA**		**Progesterone**	
	**Concentration (ng/g)** mean ± S.D. (*n*)	**Range** Min–max	**Concentration (ng/g)** mean ± S.D. (*n*)	**Range** Min–max	**Concentration (ng/g)** mean ± S.D. (*n*)	**Range** Min–max	**Concentration (ng/g)** mean ± S.D. (*n*)	**Range** Min–max	**Concentration (ng/g)** mean ± S.D. (*n*)	**Range** Min–max	**Concentration (ng/g)** mean ± S.D. (*n*)	**Range** Min–max
TYH	0.6 ± 0.3 (8)	0.3–1.1	0.3 ± 0.2 (5)	0.1–0.7	1.6 ± 1.6 (7)	0.2–4.6	0.5 ± 0.1 (8)	0.4–0.7	3.7 ± 2.8 (7)	0.7–8.9	5.7 ± 2.0 (8)	2.8–9.0
COL	2.4 ± 4.7 (11)	0.2–16.2	2.5 ± 4.0 (9)	0.1–9.5	3.0 ± 4.3 (9)	0.1–11.3	2.0 ± 4.2 (11)	0.3–14.4	4.9 ± 4.8 (8)	0.6–13.9	5.6 ± 4.6 (11)	2.0–18.8
TRO	0.6 ± 0.2 (5)	0.2–0.7	2.4 (1)	2.4	0.1 ± 0.0 (4)	0.1–0.2	0.8 ± 0.3 (5)	0.5–1.1	1.4 ± 0.9 (3)	0.4–2.2	6.1 ± 1.2 (5)	4.6–7.7
NEH	0.6 ± 0.6 (8)	0.2–1.9	0.5 ± 0.5 (5)	0.1–1.3	0.3 ± 0.2 (6)	0.2–0.6	0.5 ± 0.3 (8)	0.2–1.1	1.5 ± 1.1 (6)	0.4–2.8	5.9 ± 3.1 (8)	3.5–11.4
BLU	2.8 ± 5.6 (7)	0.5–15.4	0.3 ± 0.2 (4)	0.1–0.5	0.7 ± 0.8 (7)	0.1–2.5	0.9 ± 0.8 (7)	0.4–2.6	1.4 ± 1.0 (5)	0.2–2.4	5.1 ± 1.5 (7)	3.6–7.5

### Androgens

Testosterone was found in 95.1% of the samples and DHEA in 70.7%. Baseline concentrations (just prior to and immediately following the stress test) and the pooled sample most closely following the stress test across all five animals were, for testosterone, in the range of 0.40–0.81 ng/g dw, while maximum concentrations in animals with a discernable peak (COL, BLU) ranged from 2.57 to 14.36 ng/g dw. For DHEA, baseline concentrations were in the range of 0.55–4.28 ng/g dw, while maximum concentrations in animals with a discernable peak (COL, NEH, TYH) ranged from 2.80 to 13.92 ng/g dw.

### Progestagens

Progesterone was found in 95.1% of the samples. As expected, substantial variation in baseline progesterone was observed. Baseline concentrations (just prior to and immediately following the stress test) across all animals were in the range of 4.14–11.03 ng/g dw, while maximum concentrations in animals with a discernable peak across the remaining aggregated samples (BLU, COL, NEH, TYH) ranged from 7.51 to 18.77 ng/g dw.

### Others

A range of other hormones were observed in the epidermis samples, including 11-deoxycortisol, cortisone, estrone and androstenedione (and also traces of the male fish androgen, 11-ketotestosterone). However, the presence of these hormones was not consistent enough to let them be part of the statistical analysis.

## Discussion

### Skin renewal and sloughing rates

The bottlenose dolphins in the current study on average produced smaller volume skin samples than the physically smaller harbour porpoises that were sampled in connection with the method development (Bechshoft *et al*. 2015). Observationally, the sloughed skin that was collected was not qualitatively the same between samples. On some occasions, nearly no skin could be collected from the dolphins when scraped. On others, sloughed skin appeared as expected and on some it came off the animals in peeled sheets. It appeared as if sloughing was not a continuous process, but was possibly pulsatile with the skin collections capturing different stages of skin turnover. However, subsequent analysis found no consistent temporal pattern in the weight of the skin sampled over time. Whatever the case, the enormous variation in sloughed skin within and between individuals over the course of the study required pooling samples, which blurred the temporal resolution (across a maximum of 7 days) of the validation. Further, during hormone analysis, substantial differences were noted in skin samples with regard to texture, water content and coloration. The large variation in skin sample quantity and quality indicates the need for standardization of sampling protocols. Such a protocol would further benefit from detailing observations on how to obtain samples of sloughed skin not only from wild animals in hand in connection with tagging, disentanglement efforts or health assessments but also via remote sampling of wild cetaceans. Although remote sampling was not part of this study, it may be possible to collect sufficient samples of skin naturally sloughed by larger cetaceans at sea (Harlin *et al*. 1999; Engelhaupt 2004; Bechshoft *et al*. 2015). Skin biopsy punches from tissue banks could also be used as source material, which would enable retrospective temporal studies (Bechshoft *et al*. 2015).

Variation between subjects was broad and peaks in certain hormones, particularly those related to the classical stress response, were only notable in two of the dolphins (COL, BLU). Interestingly, these same two dolphins stood out in the stress test study described in Champagne *et al*. (2018); COL demonstrated the highest reported value of blubber cortisol at the end of the stress test (COL = 38.0 ng/g lipid), and BLU had the second highest blubber cortisol (BLU = 23.7 ng/g lipid; D. Houser, pers. com.), although level changes in response to the stress test were not reported because BLU’s baseline levels were also high. Thus, although the relationship is only observed in two animals, there is at least a correlation between the dolphins that demonstrated the highest blubber cortisol levels and the appearance of cortisol in sloughed skin samples. Since the other dolphins studied here also showed an increase in blubber cortisol across the stress test, but to a lesser degree, it might be feasible that blubber uptake of cortisol did not occur far enough to the periphery of the blubber layer so as to interface with actively proliferating margins of the dermis. It has been suggested that the amount of cortisol measured in blubber is likely dependent on the depth of the blubber biopsy and it is reasonable to expect that blubber perfusion follows a gradient such that exposure to circulating cortisol is higher closer to the deep margin of the blubber layer where vascularization is greatest (Koopman 2007; Strandberg *et al*. 2008; Trana *et al*. 2015; Champagne *et al*. 2017). Thus, the utility of using skin samples for assessing the classical stress response might depend upon the extent of perfusion into the blubber layer and over the period that serum cortisol is elevated. Unfortunately, if the extent of peripheral perfusion is critical to whether the cortisol response can be observed in skin, we cannot determine from the time course of the stress test employed by Champagne *et al*. (2018) how long an adequate perfusion might need to be for the cortisol signal to appear. It cannot be determined whether the blubber cortisol of BLU was elevated for some time prior to the stress test, or whether the blubber cortisol of COL remained elevated for some time following the stress test. Furthermore, the thickness of the skin may vary at different positions of the body. Presently, it is not known how much these parameters contribute to the variation in cetacean skin steroid concentrations.

The relationship between blubber cortisol and skin cortisol does suggest that, at least in some cases, a cortisol response to an acute stressor can be incorporated into dolphin skin. From the time lag between the stress response and the appearance of cortisol in skin, an indication of skin renewal rates can be made, assuming that the incorporation of cortisol (and other hormones) into the dolphin skin matrix is due to the rate of skin renewal once delivery of cortisol to the margin of skin turnover has occurred. Although available estimates of epidermal turnover rate ranged from 70 to 75 days (Hicks *et al*. 1985; St. Aubin *et al*. 1990), the results here suggest a lower figure of around 45–60 days. This new figure might provide a more accurate renewal rate as earlier work involved injecting the animals sub-epidermally with a labelling substance, which would require some amount of additional time to be initially incorporated in the epidermis. Factors such as solubility in lipid or water could also have affected the uptake of the substance.

### Steroid hormone measurement in cetacean skin

Laboratory analysis of the skin samples provided consistent data on androgens (testosterone and DHEA), corticosteroids (cortisol, aldosterone and corticosterone) and a progestogen (progesterone) in this matrix. A number of additional hormones were quantifiable in the skin samples from all five dolphins, albeit with a substantial degree of inconsistency due to the low volume of many of the samples: 11-deoxycortisol; cortisone; estrone; and androstenedione (and also trace elements of a fish hormone, 11-ketotestosterone). This method is also expected to be able to quantify more hormones, if larger sample sizes (100 mg dw, or more) can be processed.

### Validation of the skin as a matrix for measuring stress-related hormones

Our results demonstrate that the hormonal response to an acute stressor can be detected in the skin, although the conditions of peripheral perfusion over which it can be detected require further investigation. Baseline hormonal concentrations can be collected from skin samples immediately after the acute stressor using sloughed skin. The time-frame over which samples can be collected offers a means to detect baseline days or weeks after the stress event, in contrast to the mere minutes or hours available for pre-stressor concentrations in serum, faeces and blubber (Champagne *et al*. 2018). The known delay also provides an opportunity to assess the stress response of an animal to a stressful event long after concentrations in the other matrices have returned to baseline. It is possible that an unknown stress event may influence samples at the time of collection; however, collection and analysis of stress hormones in multiple matrices sampled at the same time (e.g. skin and blubber from a blubber biopsy) could make it possible to evaluate a wider temporal record of stress responses in the animal and thus distinguish baseline from the temporal window of the stress response. Finally, our data carries no indications of an HPA-axis homologue in the skin of the sampled dolphins (as has been found in other mammals; Slominski *et al*. 2013); if there was such a homologue, a hormone pulse would have been observed in the skin samples taken shortly after the acute stressor was experienced by the animals.

The substantial differences in inter-individual cortisol concentrations presented in this study are commonly observed in other matrices (e.g. blood and hair) too, and were also observed in our previous harbour porpoise study (Bechshoft *et al*. 2015, Bechshoft *et al*., 2012, Mislan *et al*. 2016). Although our statistical power is low with only five animals, and only three with a sufficient temporal resolution to assess the stress-to-signal time lag, controlled stress tests in cetaceans are extremely rare, making this study valuable to our understanding of the stress response and how it is reflected in different biological matrices. The results confirm and strengthen the earlier conclusion by Bechshoft *et al*. (2015) that cortisol can be extracted and measured in cetacean skin, as the total number of animals investigated now sits at nine across two species (four harbour porpoise in the development phase: Bechshoft *et al*. 2015; and five bottlenose dolphin here, in the validation phase). However, the results here also contribute to mounting evidence (e.g. Champagne *et al*. 2018) that, like cortisol and corticosterone, aldosterone is also involved in the cetacean stress response, while progesterone is not. With regards to testosterone, COL (and to a lesser degree, BLU) did produce a peak in skin of this hormone, although the dolphin stress test found testosterone to decline for several hours after the peak stress response (Champagne *et al*. 2018). Either way, it appears that the acute stress response does affect testosterone concentrations and thus may have the potential to affect reproductive effort/success. The results also suggest that DHEA may play a role in the cetacean stress response. Although observed in other species, the potential role of DHEA as stress marker is not yet well understood in marine mammals (O'Brien *et al*. 2017; Gundlach *et al*. 2018; McCormley *et al*. 2018)

This study develops and validates a non-invasive method for measuring cortisol and other hormones related to stress, health and reproduction in the skin of cetaceans. Together, these findings represent a critical step towards the use of skin samples to assess stress responses, and possibly reproductive status, in cetaceans, which could ultimately be applied as a health-assessment tool in cetaceans. The method shows potential for supporting investigations of acute and chronic stress, such as explorations of cetacean stress responses to distinct events (e.g. naval sonar exposure) or prolonged exposures (e.g. shipping noise). However, further investigation is needed to understand the magnitude of the stress response in relation to peripheral perfusion and its relationship to the skin cortisol signal, as well as baseline variations in skin hormone concentrations as affected by sex, age, reproduction, and other life-history events. Protocols for skin sampling to ensure homogenous samples of high quality and quantity are needed. Options to support this work include sampling from live and stranded animals and retrospective temporal studies, where possible (e.g. archived tissue in biobanks).

## Funding

This work was supported by The Office of Naval Research’s Marine Mammals and Biology Program (ONR MMB) [grant numbers N000141310771, N000141512187], as well as ONR MMB grants supporting the dolphin stress tests and skin sample collections [grant numbers N000141110436, N000141310770, N000141512214).
